# Computational strategy for quantifying human pesticide exposure based upon a saliva measurement

**DOI:** 10.3389/fphar.2015.00115

**Published:** 2015-05-27

**Authors:** Charles Timchalk, Thomas J. Weber, Jordan N. Smith

**Affiliations:** Health Impacts and Exposure Science, Pacific Northwest National LaboratoryRichland, WA, USA

**Keywords:** saliva, biomonitoring, salivary gland, uptake, clearance, pesticides

## Abstract

Quantitative exposure data is important for evaluating toxicity risk and biomonitoring is a critical tool for evaluating human exposure. Direct personal monitoring provides the most accurate estimation of a subject’s true dose, and non-invasive methods are advocated for quantifying exposure to xenobiotics. In this regard, there is a need to identify chemicals that are cleared in saliva at concentrations that can be quantified to support the implementation of this approach. This manuscript reviews the computational modeling approaches that are coupled to *in vivo* and *in vitro* experiments to predict salivary uptake and clearance of xenobiotics and provides additional insight on species-dependent differences in partitioning that are of key importance for extrapolation. The primary mechanism by which xenobiotics leave the blood and enter saliva involves paracellular transport, passive transcellular diffusion, or transcellular active transport with the majority of xenobiotics transferred by passive diffusion. The transcellular or paracellular diffusion of unbound chemicals in plasma to saliva has been computationally modeled using compartmental and physiologically based approaches. Of key importance for determining the plasma:saliva partitioning was the utilization of the Schmitt algorithm that calculates partitioning based upon the tissue composition, pH, chemical pKa, and plasma protein-binding. Sensitivity analysis identified that both protein-binding and pKa (for weak acids and bases) have significant impact on determining partitioning and species dependent differences based upon physiological variance. Future strategies are focused on an *in vitro* salivary acinar cell based system to experimentally determine and computationally predict salivary gland uptake and clearance for xenobiotics. It is envisioned that a combination of salivary biomonitoring and computational modeling will enable the non-invasive measurement of chemical exposures in human populations.

## Introduction

The National Research Council of the National Academies report, *Toxicity Testing in the 21st Century: A Vision and Strategy*, highlighted the importance of quantitative exposure data for evaluating human toxicity risk (Hubal, 2009). The report supports the use of chemical exposure data to provide critical information on the magnitude, timing, and duration of a biologically relevant dose delivered to target tissues (Sheldon and Cohen Hubal, 2009; Cohen Hubal et al., 2010). Direct measurement of chemical exposures using personal monitoring provides the most accurate estimation of a subject’s true exposure (Nieuwenhuijsen et al., 2006). In this context, biomonitoring is a critical tool for quantitatively evaluating exposure from both environmental and occupational settings to a wide range of pollutants, including pesticides (Borzelleca and Skalsky, 1980; Nigg and Wade, 1992; Friberg and Elinder, 1993; Christensen, 1995; Angerer et al., 2006, 2007; Barr et al., 2006). Non-invasive methods have also been advocated for quantifying the pharmacokinetics and bioavailability of drugs and xenobiotics, and the use of saliva has been suggested as an ideal body fluid that can be substituted for blood in biomonitoring (Pichini et al., 1996; Timchalk et al., 2004; Timchalk et al., 2007b). In this regard, a broad range of drugs, organic chemicals, metals, and pesticides are readily secreted in saliva (Borzelleca and Skalsky, 1980; Drobitch and Svensson, 1992; Nigg and Wade, 1992; Lu et al., 1997, 1998; Silva et al., 2005). For many of these xenobiotics, saliva concentration readily correlates with blood concentration; hence, it is feasible to utilize pharmacokinetic models to accurately estimate systemic dose based upon a saliva measurement.

**FIGURE 1:**
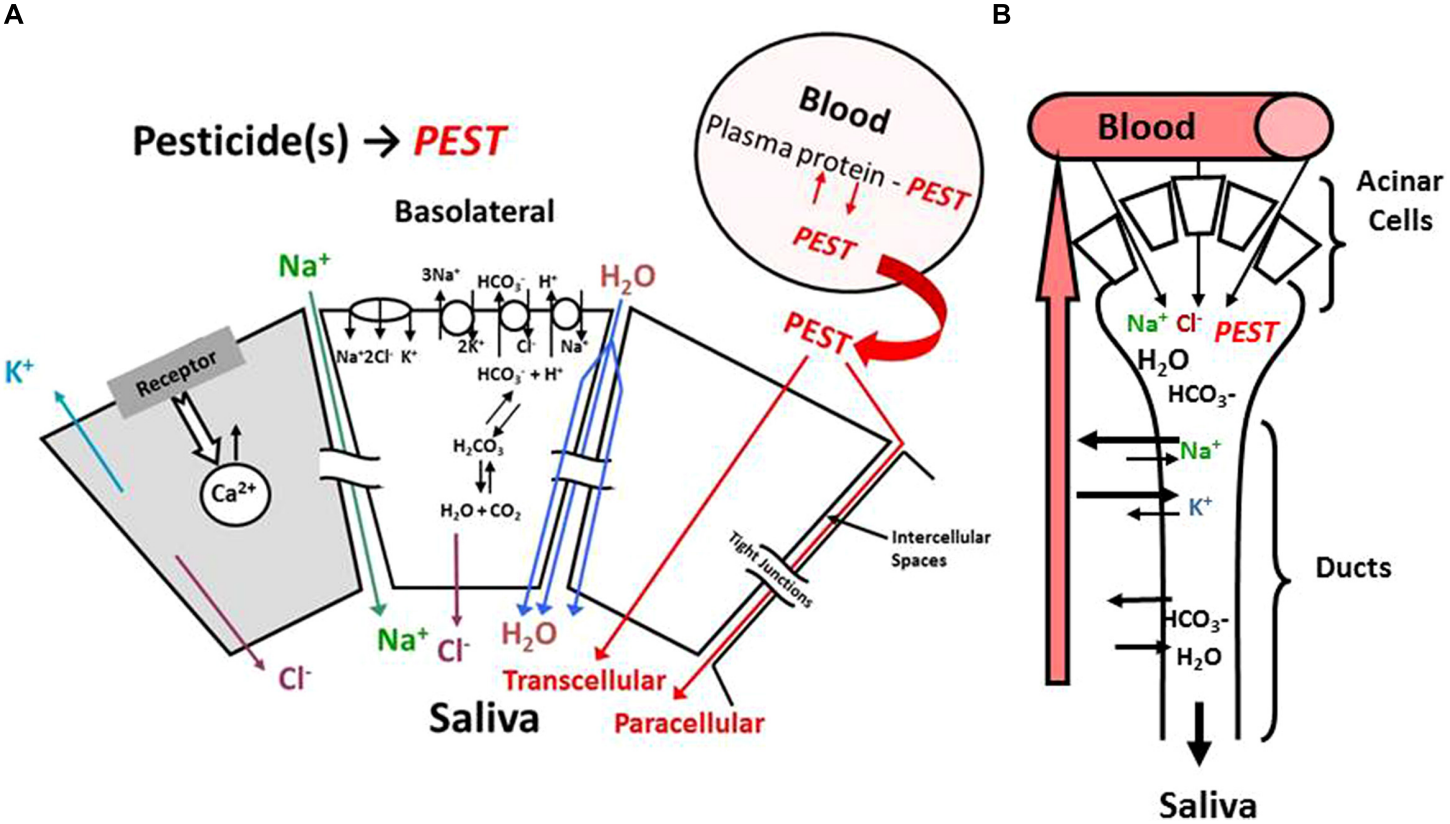
**Schematic model illustrating (A) acinar cell and (B) salivary duct function associated with saliva formation**. PEST is used as a general notation for pesticides.

The mechanism of acinar cell activation, electrolyte transport and saliva formation illustrated in **Figure 1** is derived from an extensive literature that has characterized these processes in detail (Nauntofte, 1992). Salivary glands are comprised of a number of major (parotid, submandibular, and sublingual) and minor glands that primarily consist of acinar cells that collectively produce saliva (for review, see Hold et al., 1995b). Salivary glands are highly perfused and blood flow is in a countercurrent direction to salivary flow (**Figure 1B**; Davenport, 1977). The primary mechanism by which xenobiotics leave the blood and enter saliva is thought to involve paracellular transport, passive transcellular diffusion, or transcellular active transport (**Figure 1A**; Landon and Mahmod, 1982). Paracellular transport (i.e., ultrafiltration) favors small (MW ∼300 Da) polar lipid insoluble molecules that generally have a low (i.e., <1.0) saliva/plasma (S/P) ratio. Whereas, transcellular diffusion or active transport are favored by lipid soluble materials that can readily cross cell membranes (Hold et al., 1995a). The majority of drugs and xenobiotics are cleared from plasma into saliva by a passive diffusion process that is a function of concentration gradient, surface area, membrane thickness, and diffusion constants (Hold et al., 1995b). This focused review will describe how the modeling of this passive diffusion process in both rats and humans was accomplished utilizing a pharmacokinetic model that were initially developed for the organophosphorus insecticide chlorpyrifos (*CPF*) and its major metabolite trichloropyridinol (*TCPy*; Smith et al., 2010, 2012).

It is well recognized that biomonitoring offers one of the best strategies for quantifying human dosimetry and assessing risk associated with both occupational and environmental chemical exposures (Friberg and Elinder, 1993; Christensen, 1995). As noted above, saliva has been suggested as an ideal non-invasive body fluid, yet there are a number of limitations which have hampered saliva’s use in biomonitoring. First, to utilize saliva for biomonitoring the relationship between blood and saliva concentrations for target biomonitoring, analytes needs to be well established (Timchalk et al., 2004). Second, to more broadly exploit saliva as a matrix it is critical to *a priori* identify which chemicals are readily cleared in saliva at levels that can be quantified analytically. The current manuscript reviews the initial saliva model development focused on *in vivo* uptake and clearance studies in the rat as well as computational prediction in humans (Smith et al., 2010, 2012). In addition, a partitioning coefficient algorithm that readily accommodates species-dependent differences in plasma protein-binding and pH is presented. This is of particular importance since changes in binding and/or pH appear to have a significant impact on plasma:saliva partitioning thereby contributing to variable saliva analyte concentrations. Finally, a brief perspective on the utility of cell based *in vitro* assay systems and computational modeling are presented as a potential strategy for broad based screening of chemicals for their salivary clearance potential (Weber et al., 2015).

## Computational Methods

### Pharmacokinetic Modeling Strategy

**FIGURE 2:**
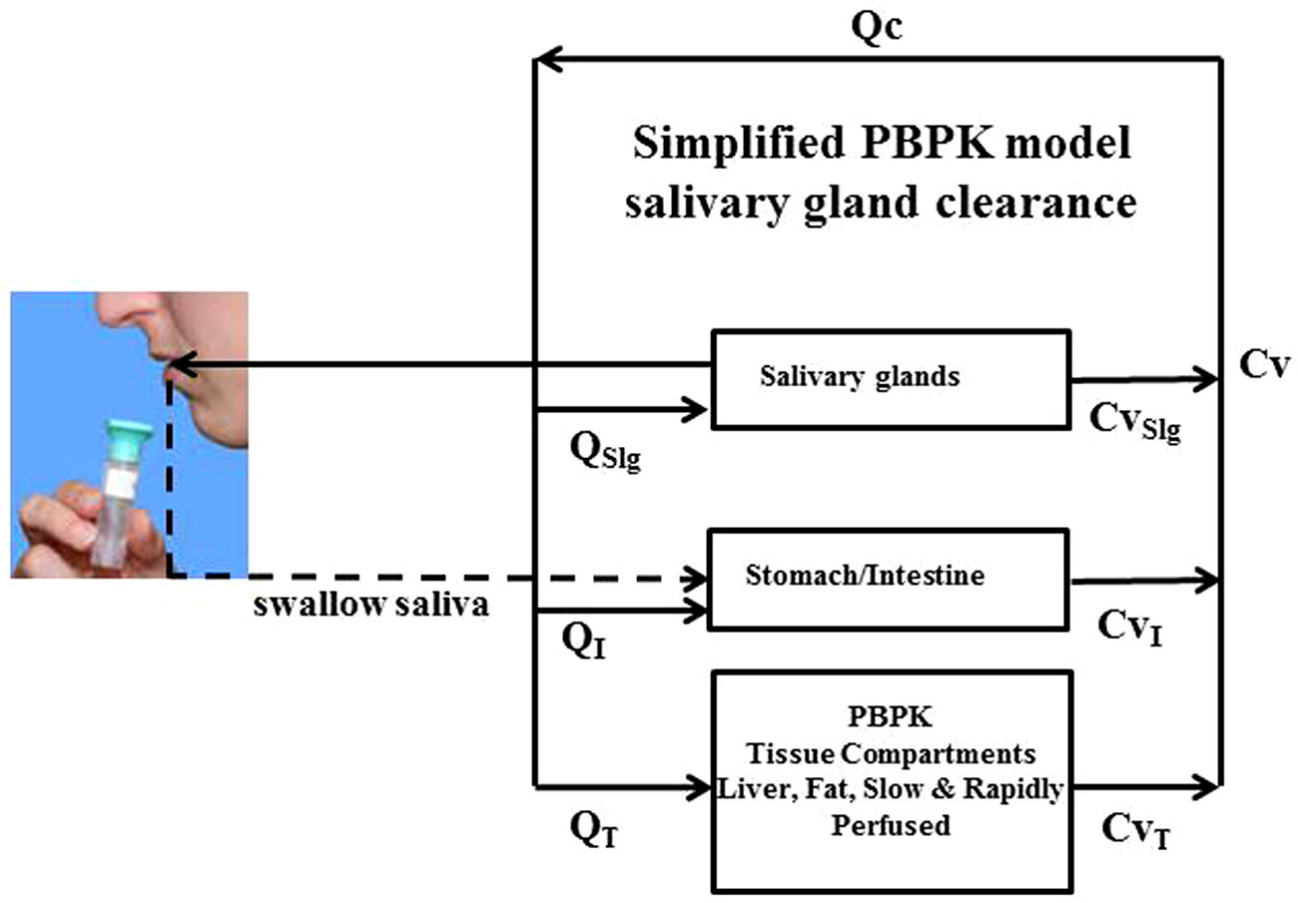
**Schematic model illustrating physiologically based pharmacokinetic (PBPK) model structure for simulating *in vivo* salivary gland clearance of pesticides**.

The overarching computational modeling approach utilizes a physiologically based pharmacokinetic (PBPK) model as illustrated in **Figure 2**. Within this PBPK model both the *CPF* and *TCPy* blood to saliva pharmacokinetics in the rat was handled as a simple one-compartment pharmacokinetic model as previously described (Smith et al., 2012). The model was further modified to enable the prediction of *CPF* and *TCPy* concentrations in human saliva after simulated exposures, by accommodating differential partitioning based upon species dependent differences in pH (see below for details). In this model, *CPF* and it neurotoxic metabolite chlorpyrifos-oxon (*CPF-oxon*) were distributed among internal compartments mediated by blood circulation described as a flow-limited process. The fraction of *CPF* or *TCPy* that was not bound to plasma-protein is described simply as a fraction bound, where FB*_CPF_* or FB*_TCPy_* are the fractions of *CPF* or *TCPy* bound to plasma-proteins, C*_CPF_*_b_ and C*_TCPy_*_b_ are the concentration of *CPF* or *TCPy* bound to plasma proteins, and C*_CPF_*_bl_ and C*_TCPy_*_bl_ are the total concentration of *CPF* or *TCPy* in blood, respectively (Eqs 1 and 2). The free *CPF* and *TCPy* concentrations (i.e., C*_CPF_*_f_ and C*_TCPy_*_f_) are available to partition to various tissue compartments based upon their tissue specific partition coefficients (Timchalk et al., 2002). Fractional plasma protein-binding of *CPF* and *TCPy* were measured previously and both determined to be ∼98–99% bound (Lowe et al., 2009; Smith et al., 2010). As *CPF-oxon* undergoes rapid hydrolysis in the presence of albumin, it was assumed that *CPF* and *CPF-oxon* have equivalent fraction binding in plasma. Human metabolic parameters, including *CPF* dearylation, *CPF* desulfuration, and *CPF-oxon* hydrolysis, were updated (Smith et al., 2011). Other human parameters were also updated from a variety of literature sources, including cholinesterase activities, enzyme aging, degradation, reactivation, and turnover rates (Sidell and Kaminskis, 1975; Hojring and Svensmark, 1976; Maxwell et al., 1987; Mortensen et al., 1998; Li et al., 2005; Albers et al., 2010; Smith et al., 2011). To accommodate the effects of isoflurane anesthesia (required during *in vivo* experiments) on rat physiology, the cardiac output was reduced 15% from standard values based upon observed *in vivo* effects [15 × body weight (kg)^0.75^; Conzen et al., 1992; Vollmar et al., 1992; Brown et al., 1997].

$$CPF:C_{CPF}{}_{f}\;=\;C_{CPF}{}_{\mathrm{bl}}\;\times \;{\mathrm{FB}}_{CPF}\mathrm{(1)}$$

$$TCPy:\;C_{TCPy}{}_{f}\;=\;C_{TCPy}{}_{\mathrm{bl}}\;\times \;{\mathrm{FB}}_{TCPy}\mathrm{(2)}$$

### Saliva Partitioning

In the current PBPK model structure, transport of *CPF* or *TCPy* from blood to saliva is modeled as a passive diffusion process based upon their respective partitioning. Briefly, for both analytes the saliva concentration (C*_sal_*) was defined as their blood concentration (C*_bl_*) multiplied by the saliva/blood partitioning coefficient (P*_sal/bl_*) [Eq. 3], which was determined experimentally (in rats) using the mean saliva/blood concentration ratio.

$$C_{sal}=C_{bl}\times P_{sal/bl}$$

However, due to experimentally observed saliva pH changes in rats (8.5–9.5; Smith et al., 2010), and the reported variability in saliva pH between humans (6.2–7.4), species differences may exist for ionizable compounds with pKa/pKb values near physiologically relevant pH ranges. Thus in the absence of human-specific experimental data, algorithms can be useful for estimating partitioning and/or extrapolating partitioning measured in animal models. Numerous partitioning algorithms exist (Poulin and Krishnan, 1995; Rodgers et al., 2005; Schmitt, 2008; Peyret et al., 2010). Here, the Schmitt algorithm was utilized, because it is the only algorithm that has been previously validated for predicting salivary partitioning measured in rats (Schmitt, 2008; Smith et al., 2010). The approach calculates partition coefficients based upon the tissue composition in terms of water, lipid and phospholipid contents, proteins, pH differences using chemical compound-specific lipophilicity, pKa, as well as plasma protein-binding. As noted in Smith et al. (2010), this algorithm assumes that ionized compounds are capable of traversing membranes, although at a much lower rate (∼3-orders of magnitude) than non-ionized forms and estimates the difference in rates based upon the ratio of distribution coefficients for the ionized and unionized forms (Schmitt, 2008).

The Schmitt algorithm was adapted for use in saliva (Schmitt, 2008; Smith et al., 2010). This original approach did not consider salivary partitioning, thus “tissue-specific” parameters were used to make these predictions with saliva (**Table 1**). Otherwise the Schmitt algorithm was used as described (Schmitt, 2008). Briefly, for each of the model compartments, the unbound fraction (*f_u_*; Eq. 4) is the ratio of analyte unbound concentration (*C_u_*) over total concentration (*C_total_*). Generally, it is thought that neutral compounds cross biological membranes much more easily than charged compounds. Thus pH gradients that exist across membranes can have a profound influence on the ability of ionizable compounds to cross that barrier, where the ratio of ionized to neutral compounds can vary by pH and the pKa of the compound. For example, a week acid can accumulate of the side of the barrier that has the higher pH, since the ionized form cannot cross the membrane as easily as the uncharged compound. The equilibrium salivary gland:blood (*P_sal:bl_*) partitioning coefficient is based upon the relationships expressed in Eq. 5; where *C_t_*, *C_p_*, *C_int_*, and *C_cell_* are the total concentrations in saliva tissue, plasma, interstitial, and cellular spaces, respectively. The volume fractions of interstitial and cellular space are *F_int_* and *F_cell_* while the unbound fractions in interstitial, cellular and plasma are f_u_^int^,f_u_^cell^, and f_u_^p^, respectively. The unbound fraction in the interstitial space is estimated relative to the unbound fraction in plasma, based on the fact that the interstitial fluid is quite similar to plasma but with lower concentrations of proteins and lipids (Schmitt, 2008); whereas the unbound fraction in the cellular space is determined by chemical affinity for cellular macromolecules such as protein and lipids (both neutral and charged phospholipids). Finally, the tissue pH gradients are critical since they will influence the unbound concentrations for weak acids and bases which are charged under physiological conditions. This is of particular importance when considering salivary gland clearance where saliva formation results in acinar cell acidification (see **Figure 1**) as a result of carbonic anhydrase conversion of CO_2_ and H_2_O to HCO_3_^-^ and H^+^ (Nauntofte, 1992).

$$f_{u}=\frac{C_{u}}{C_{total}}\mathrm{(4)}$$

$$P_{sal:bl}\;=\frac{C_{t}}{C_{p}}=\frac{C_{t}}{C_{u}}\frac{C_{u}}{C_{p}}=\frac{F_{int}C_{int}+F_{cell}C_{cell}}{C_{u}}\frac{C_{u}}{C_{p}}\;45pt=\left(\frac{F_{int}}{f_{u}^{int}}+\frac{F_{cell}}{f_{u}^{cell}}\right)f_{u}^{p}\mathrm{(5)}$$

**Table 1:** Input parameters for a modified algorithm for calculating the saliva:blood trichloropyridinol (*TCPy*) partitioning coefficient (based upon Schmitt, 2008).

Parameter	Value (rat/human)	Source (rat/human)
**TCPy physiochemical properties**		
Fraction unbound in plasma	0.015	Measured/estimated
pKa	4.55	Fixed^a^
Log *K*_ow_ at 7 pH	1.3	Fixed^a^
Log *K*_ow_ at 3 pH	3.2	Fixed^a^
α	0.013	Calculated^b^
**Tissue properties**		
** Plasma**		
Fraction protein	0.073	Fixed^c^
Fraction water	0.915	Fixed^c^
pH	7.8/7.4	Measured/fixed
**Saliva**		
Fraction cells	0	Estimated^d^
Fraction protein	0.003	Fixed^c^
Fraction water	0.98	Fixed^c^
pH	8.9/6.7	Measured/fixed^c^

*^a^Racke (1993) and Shemer et al. (2005)^b^α ratio of *K*_ow_ at pH 7 and 3 (Schmitt, 2008) ^c^Ritschel and Tompson (1983) and Hold et al. (1995b)^d^Assume cellular fraction of saliva negligible*.

Parameter inputs (**Table 1**) for this algorithm include: physical/chemical properties of *CPF* and *TCPy*, physiological properties of rat and human plasma and mixed saliva; additional assumptions included pH dependent partitioning to the interstitial space fraction and negligible cellular fraction of saliva (Ritschel and Tompson, 1983; Racke, 1993; Hold et al., 1995b; Shemer et al., 2005).

### Salivary Flow Rate

To experimentally obtain adequate saliva from rats, animals were administered a cholinergic agonist, pilocarpine to induce significant salivation (Timchalk et al., 2006; Smith et al., 2010). To facilitate a more accurate model simulation of this physiological response, the salivary flow rate (Q*_sal_*) was defined as a non-linear dynamic equation where A, B, and C are constants used to fit salivary flow rate data (Smith et al., 2010) from rats infused (3 ml/h) with pilocarpine (1 mg/ml; Eq. 6).

$$Q_{sal}(t)\;=\;A\;\times \;t^{B}\;+\;\mathrm{C (6)}$$

The *CPF* or *TCPy* elimination rates in saliva were defined as their respective concentrations in saliva (C*_sal_*) multiplied by the saliva flow rate (Eq. 7). However, PBPK model simulations of humans did not accommodate differences in saliva flow rates.

$$\frac{dC_{sal}}{dt}=-C_{sal}\;\times \;Q_{sal}\mathrm{(7)}$$

### Sensitivity Analysis

To provide additional insight into the relative importance of model parameters, as part of this review, a local sensitivity analysis was conducted to identify the most important parameters for estimating the plasma:saliva partitioning coefficient for a generic compound with 50% plasma-protein binding, pKa = 7.0, log *K*_ow_ (non-ionized) = 2 and log *K*_ow_ (ionized) = -1. The analysis focused on the impact of changes in these parameters on the partitioning coefficient at steady-state. The normalized sensitivity coefficients were calculated for a 1% change in a given model parameter when all other parameters were held fixed. Sensitivity analysis was performed using the forward difference method coded in acslX version 3.0.2.1 (AEgis Technologies, Inc., Huntsville, AL, USA).

## Saliva Model Development Review

**FIGURE 3:**
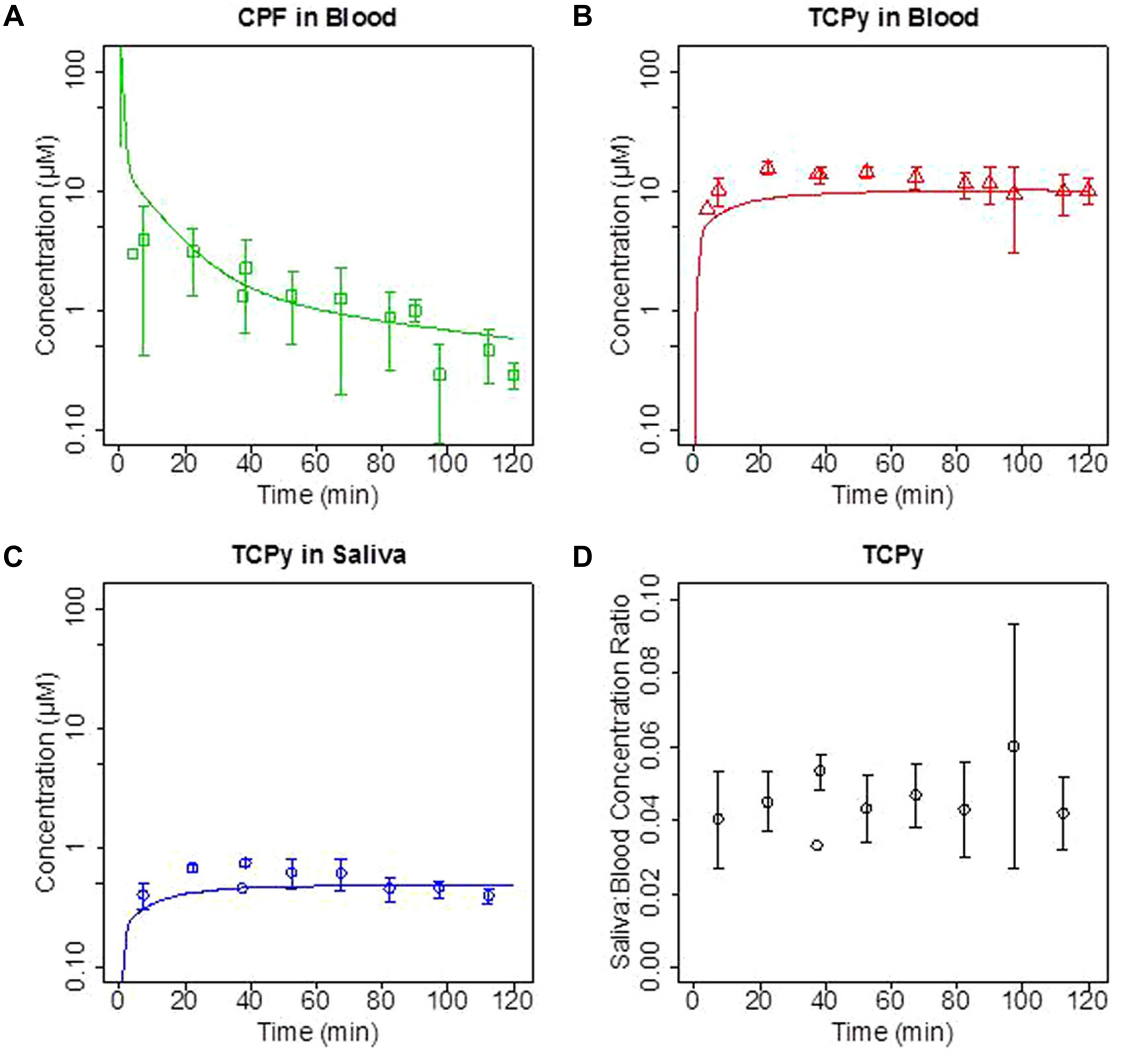
**Groups of rats were administered a single intravenous (IV) dose of 5 mg/kg chlorpyrifos (*CPF*). (A)** Concentration (μM) of *CPF* in blood; **(B,C)** trichloropyridinol (*TCPy*) in blood and saliva, respectively; and **(D)** saliva:blood *TCPy* concentration ratios from 10–120 min post-dosing. Lines are PBPK model fit to data.

Initial *in vivo* studies in rats focused on the comparison of blood and saliva *TCPy* concentration time-course following intravenous (IV) administration of the *CPF* over a broad dose range (0.5–5 mg/kg). These *in vivo* experiments focused on exposing rats to *CPF* by IV administration and quantifying the time-course of *CPF* and *TCPy* in both blood and saliva over a range of *CPF* doses. Previous studies have characterized route-dependent pharmacokinetic differences in rats, and IV administration was chosen for these studies to minimize first-pass hepatic metabolism thereby maximizing the concentration of *CPF* in blood available for salivary clearance (Busby-Hjerpe et al., 2010). These previous studies demonstrated that the blood:saliva ratios were not impacted by a range of factors including: *CPF* or *TCPy* dose, pilocarpine dose, or the timing of saliva collection (Smith et al., 2010, 2012). In **Figures 3A–C** where the time-course of *CPF* (blood) and it major metabolite *TCPy* (blood and saliva) are illustrated following a 5 mg/kg IV dose. These results are very consistent with previous observations that *CPF* undergoes rapid metabolism to *TCPy* (Poet et al., 2003; Timchalk et al., 2007a; Busby-Hjerpe et al., 2010), although *CPF* was detectable in three saliva samples (data not shown). Results (**Figure 3D**) were also consistent with previous *TCPy* dosing experiments where the ratio of *TCPy* in saliva/blood (ratios ∼0.0 4–0.06) demonstrated AUC linearity consistent with parallel pharmacokinetics of *TCPy* in blood and saliva. These initial *in vivo* studies in rats clearly demonstrate that saliva analyte concentrations are highly proportional to blood concentrations and PBPK model simulations accurately predict saliva/blood concentrations over a range of doses, consistent with a diffusion based transport mechanism.

Although in the rat *in vivo* model system saliva/blood *TCPy* concentration ratios did not change over varying experimental conditions (**Figure 3D**), as previously mentioned normal differences in saliva pH values for humans (range 5.6–7.9) have been noted in the literature (Hold et al., 1995b). Hence, to adequately accommodate differences in partitioning due to variable pH, and to enable accurate rat to human extrapolation it was critical to simulate changes in analyte ionization states particularly when the pKa was bracketed by the pH range of saliva. In this regard, the Schmitt algorithm provided a good model prediction of the saliva/blood partitioning (Smith et al., 2010). These initial finding do suggest that inter- and intra-species differences in blood:saliva partitioning may be of importance. To further evaluate the potential impact of these differences a model sensitivity analysis was conducted.

### Sensitivity Analysis

Model simulations and a sensitivity analysis was conducted to further evaluate the impact of variable pKa (4→10) or protein-binding fractions (0.1→0.9) on the saliva:blood partitioning coefficient. A summary of model parameters for both rat and human are presented in **Table 2**. For the model sensitivity analysis, it was assumed that generic compounds were 50% bound to plasma proteins, the log *K*_ow_ was 2 for non-ionized compounds, and the log *K*_ow_ was -1 for ionized compounds. The sensitivity analysis (**Table 3**) specifically focused on four model parameters that modulate the plasma:saliva partitioning coefficient, these included: plasma protein-binding, *K*_ow_ (non-ionized and ionized) and pKa. For this analysis, the model is highly sensitive [normalized sensitivity coefficient (SC) >0.5] to changes in plasma protein-binding (SC- 2.0) and pKa (-0.6 and 0.9) for both humans and rats. Hence, these parameters are of critical importance and can substantially impact partitioning.

**Table 2:** Input chemical and tissue parameters used to simulate the saliva:blood partitioning for generic over a range of pKa and protein-binding values.

Parameter	Value	Source
Fraction unbound in plasma	0.1, 0.5, or 0.9	Fixed
PKa	4,7, or 10	Fixed
Log *K*_ow_ @ non-ionized	2	Fixed
Log *K*_ow_ @ ionized	-1	Calculated from α
α	0.001	Schmitt (2008)
**Tissue parameter**	**Value (rat/human)**	**Source**
** Plasma**		
Fraction protein	0.073	Ritschel and Tompson (1983), Hold et al. (1995b)
Fraction water	0.915	Ritschel and Tompson (1983), Hold et al. (1995b)
pH	7.4	Smith et al. (2010)
** Saliva**		
Fraction cells	0	Estimated
Fraction protein	0.003	Ritschel and Tompson (1983), Hold et al. (1995b)
Fraction water	0.98	Ritschel and Tompson (1983), Hold et al. (1995b)
pH	8.9/6.7	Ritschel and Tompson (1983), Hold et al. (1995b), and Smith et al. (2010)

**Table 3:** Sensitivity analysis for selected parameters for generalized compound plasma:saliva partitioning coefficent.

Sensitivity coefficient (SC)				
**Species**	**Fraction unbound in plasma**	***K*_*ow*_ non-ionized**	***K*_*ow*_ ionized**	**pKa**
Human	2.0	1.9 10^-4^	1.6 10^-2^	0.9
Rat	2.0	2.2 10^-4^	3.9 10^-3^	0.6

*Absolute values for SC 0.5 suggest that model is highly sensitive to this parameter. Model simulations were run at 50% protein-binding and pKa = 7.0, *K*_*ow*_ values from [T2]**Table 2***.

**FIGURE 4:**
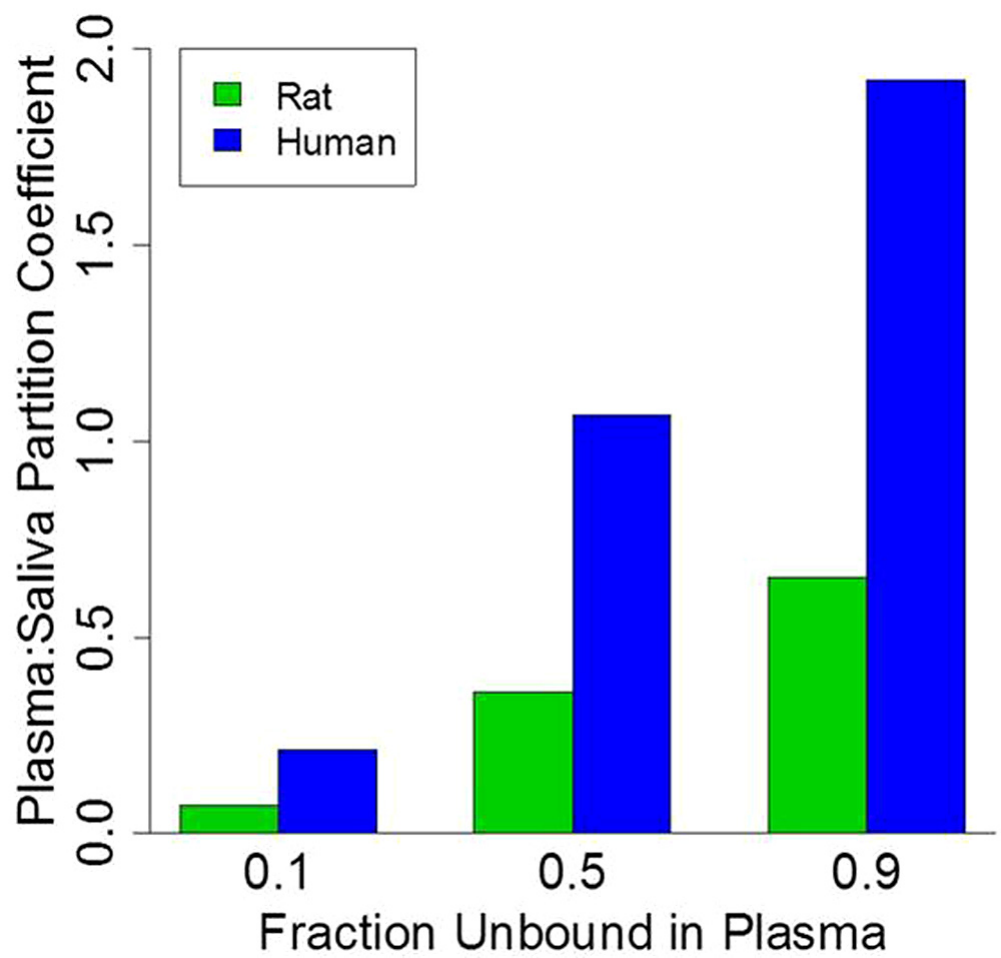
**Physiologically based pharmacokinetic model simulation of plasma:saliva partitioning coefficients for three generic compounds with varying plasma protein-binding**.

To illustrate the impact of plasma protein-binding on the saliva:plasma partitioning coefficient, model simulations were conducted for three generic compounds with 0.1, 0.5, and 0.9 unbound fraction in plasma of rats and humans. It was assumed that generic compounds had a pKa of 7, the log *K*_ow_ was 2 for non-ionized compounds, the log *K*_ow_ was -1 for ionized compounds, and salivary pH was 6.7 and 8.9 for humans and rats, respectively (**Table 2**). Model simulations are presented in **Figure 4**. For both rats and humans the plasma:saliva partitioning coefficient increases as a function of the unbound chemical fraction in plasma; however, it is also of interest to note that the partitioning is substantially greater in humans (2.7–3.3x) than in rats.

**FIGURE 5:**
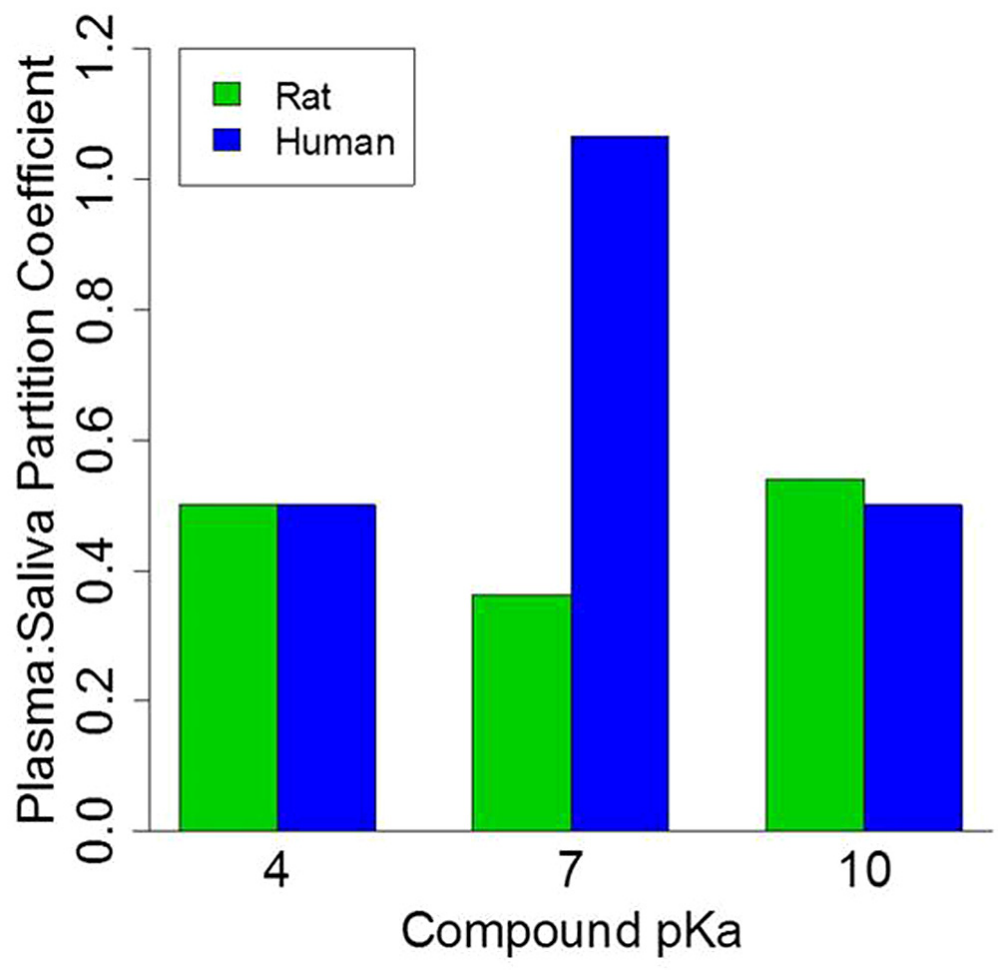
**Physiologically based pharmacokinetic model simulation of plasma:saliva partitioning coefficients for three generic compounds with varying *pKa* values**. Protein binding set at 50%.

To further explore the impact of pH on the salivary:plasma partition coefficients a similar simulation was run but in this case the protein-binding was assumed to be 50% while the pKa values were 4, 7, and 10. As illustrated in **Figure 5**, generic model simulations indicate that for those chemicals with pKa of ∼7 the partitioning coefficient will vary by a factor of 3 between rats and humans with substantially greater partitioning in humans. However, for chemicals with low or high pKa values (relative to pH) the rat and human plasma:saliva partitioning are comparable. It is also important to note that inter-individual differences is the extent of plasma protein-binding and saliva pH within a population would also contribute to variations in the saliva:plasma partitioning (data not shown); which could result in variable salivary clearance.

## Discussion

Physiologically based pharmacokinetic models have been developed that incorporated saliva clearance as a computational tool to quantify non-invasive biomonitoring of heavy metals and pesticides (Timchalk et al., 2001, 2007b; Barry et al., 2009; Smith et al., 2010, 2012). As previously noted, transcellular diffusion (**Figure 1**) is thought to be the primary method by which many drugs, chemicals, and pesticides, including the *CPF* metabolite *TCPy* transfer from blood to saliva (Haeckel, 1993; Jusko and Milsap, 1993; Lu et al., 1997, 1998, 2003; Smith et al., 2010, 2012). As noted in **Figure 3D**, *in vivo* partitioning of *TCPy* from blood to saliva in rats is very constant over a range of varying conditions which is entirely consistent with a partitioning mechanism associated with transcellular diffusion as proposed by Smith et al. (2010, 2012).

When considering transcellular or paracellular diffusion, it is of interest to note that a number of physiochemical properties including molecular size, lipid solubility, the dissociation constant of ionized compounds, and the extent of plasma-protein binding all contribute to modifying the diffusion across a concentration gradient from blood to saliva. In the current model simulation (**Figure 3**) the analyte concentration in saliva (based upon partitioning) is directly reflective of the concentration that is not bound to plasma proteins in blood, since protein-analyte complexes are too large for transcellular/paracellular diffusion (Haeckel, 1993; Jusko and Milsap, 1993; Lu et al., 1998; Smith et al., 2012).

Likewise, as shown in **Figure 5**, the pH of blood and saliva are important parameters modulating salivary clearance of compounds particularly where the chemical pKa is comparable to the pH of biological fluids (Haeckel, 1993; Jusko and Milsap, 1993). Although blood pH is reasonably consistent (pH 7.4), saliva pH can vary and is primarily controlled by the amount of bicarbonate present in saliva (Haeckel, 1993). Hence, due to variation in rat and human pH values (Smith et al., 2010), the Schmitt algorithm (Schmitt, 2008) has been utilized successfully to calculate plasma:saliva partitioning. This modification enables the extrapolation of the blood to saliva analyte partitioning coefficient to humans over a range of reported human salivary pH levels.

Although transcellular or paracellular diffusion across tight junctions has been noted as a key transport mechanism for many drugs and environmental contaminants (Haeckel, 1993; Jusko and Milsap, 1993), both active and carrier-assisted cellular transport may be of importance for some xenobiotics. In this regard, transport is dependent upon specific rate-limiting transport mechanisms. In salivary glands, unidirectional and saturable influx of amino acids, neurotransmitters, and drugs have been shown to follow classic Michaelis–Menten kinetics where affinity and velocity constants can be experimentally derived and computationally solved (Eq. 8). In this scenario, *T_r_* is the transport rate, *T_max_* the maximum rate of transport, *C_u_* the unbound analyte concentration and *K_m_* is the transport affinity constant. Hence, the current model structure can be readily modified to accommodate these active transport processes; thereby, enabling the models to simulate salivary clearance for a broad range of chemicals and drugs.

$$T_{r}=\frac{T_{\max}C_{u}}{K_{m}+C_{u}}\mathrm{(8)}$$

**FIGURE 6:**
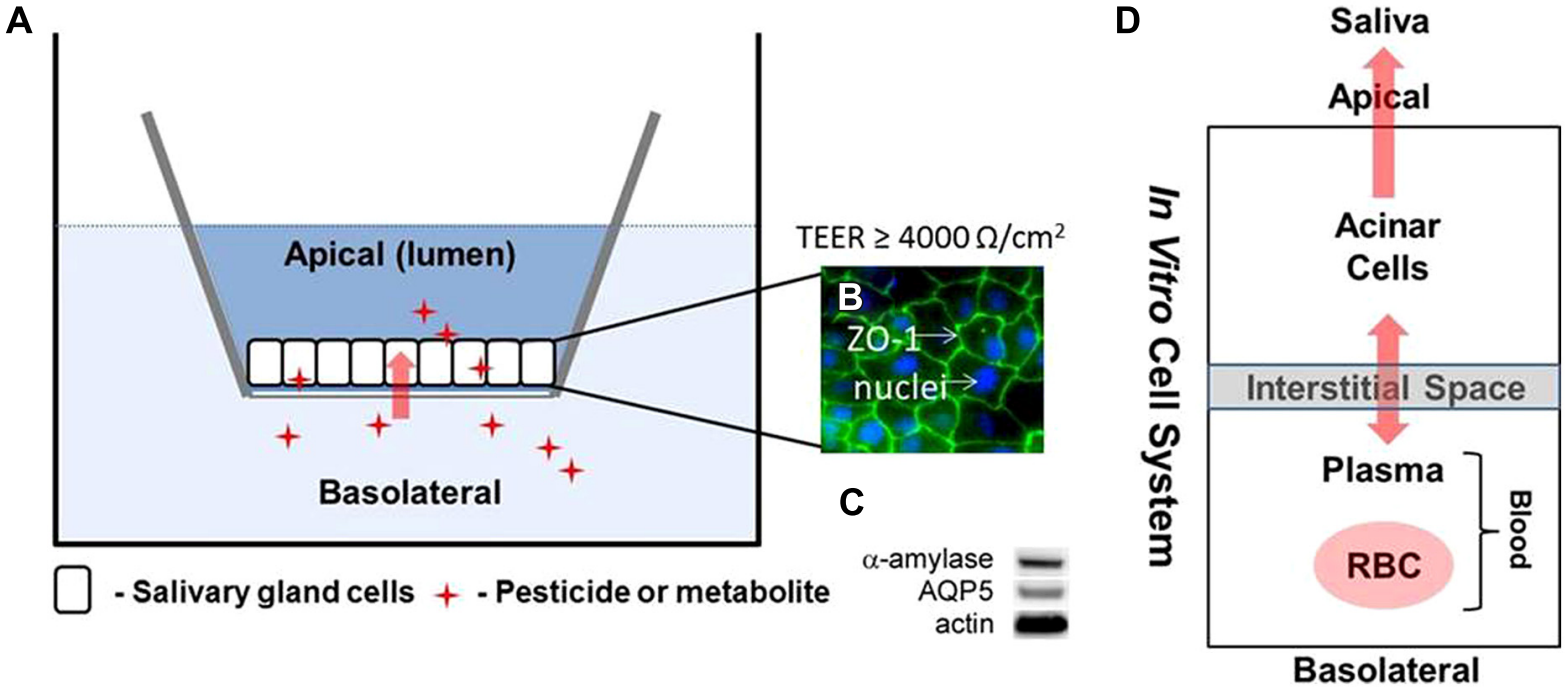
**Schematic model illustrating **(A,B)** Transwell^®^ with salivary gland epithelial cell (SGEC) system**. The tight junction marker zona occludins-1 (ZO-1) localizes to points of cell–cell contact in association with excellent tight junction formation as indicated by measurements of transepithelial electrical resistance (TEER). **(C)** Western blot analysis showing expression of α-amylase and AQ5 proteins in SGEC lysates indicative of acinar cells; and **(D)** four-compartment (blood: RBC/plasma and salivary gland: interstitial and cellular space) model structure for calculating steady-state transport.

For saliva biomonitoring to be more broadly utilized there is a need to rapidly identify a comprehensive range of chemical and drugs that can readily be quantified in saliva and utilized to predict systemic dose based upon these saliva measurements. A major limitation of the current experimental and modeling strategy is the dependence upon *in vivo* animal model systems as a means of identifying and screening chemical/drug candidates for salivary clearance. In this regard, the current *in vivo* models are limited by relatively low throughput and experimental complexity. To address these limitations we are developing an *in vitro* salivary gland epithelial cell based Transwell^®^ assay to enable broad based screening of uptake and clearance mechanisms associated with both diffusional and active transport mechanisms (**Figure 6**; Weber et al., 2015). Initial characterization of the cell system has demonstrated that tight junctions are well formed and cells maintain functional characteristics (**Figure 6C**) associated with salivary acinar cells. The computational approach is based on a simplified four-compartment model **Figure 6D**) exploiting the Schmitt algorithm (Schmitt, 2008) to describe diffusional based processes along with active transport (Eq. 8). It is anticipated that this novel experimental and computational strategy will enable the prediction of chemical uptake and clearance in saliva for a broad range of chemicals based upon limited *in vitro* experiments which are integrated into the pharmacokinetic modeling framework.

## Conclusion

Biomonitoring is a critical tool for quantitatively evaluating exposure from both environmental and occupational settings and saliva has been advocated as a potentially important non-invasive method that could be substituted for blood or urine. However, there are a number of limitations that have hampered saliva’s use in biomonitoring, including the need to initially identify which chemicals are readily cleared in saliva at concentrations that can be quantified. This review describes recent advances in the use of a computational modeling approach that is closely coupled to *in vivo* and *in vitro* experiments to predict salivary uptake and clearance of xenobiotics. The approach simulates transcellular or paracellular diffusion of unbound chemicals in plasma to saliva using a combination of compartmental and physiologically based computational models. Of key importance for determining the plasma:saliva partitioning is the utilization of a modified Schmitt algorithm (Schmitt, 2008) that calculates partitioning based upon the tissue composition, pH, chemical pKa, and plasma protein-binding. Sensitivity analysis of key model parameters specifically identified that both protein-binding and pH/pKa had the most significant impact on the determination of partitioning and that there were clear species dependent differences based upon physiological variance between rats and humans. Future research needs to focus on extending this modeling strategy to an *in vitro* salivary acinar cell based system that can be utilized to experimentally determine and computationally predict salivary gland update and clearance for a broad range of xenobiotics. Hence, it is envisioned that a combination of salivary biomonitoring and computational modeling will enable the non-invasive measurement of both environmental and occupational exposure in human populations.

## Conflict of Interest Statement

Dr. Charles Timchalk and Dr. Jordan N. Smith have previously received funding from The Dow Chemical Company, the manufacturer of chlorpyrifos (CPF), to conduct research. However, the Dow Chemical Company had no input on the current research.
